# Treatment of Pediatric Intercondylar Humerus Fracture With External Fixation and Percutaneous Pinning After Closed Reduction

**DOI:** 10.3389/fped.2022.916604

**Published:** 2022-07-11

**Authors:** Wen Shu, Rong Zhao, ZiMo Yang, XiangRui Li, GuoYong Jiang, Saroj Rai, Haobo Zhong, Xin Tang

**Affiliations:** ^1^Department of Trauma Orthopaedics, Liuzhou People’s Hospital, Liuzhou, China; ^2^Tongji Medical College, Wuhan Union Hospital, Huazhong University of Science and Technology, Wuhan, China; ^3^Department of Orthopedics and Trauma Surgery, Karama Medical Center, Dubai, United Arab Emirates; ^4^Department of Orthopaedics, Huizhou First Hospital, Huizhou, China; ^5^Department of Orthopaedic Surgery, Tongji Medical College, Union Hospital, Huazhong University of Science and Technology, Wuhan, China

**Keywords:** closed reduction, external fixation, intercondylar fracture, distal humerus, children

## Abstract

**Background:**

It is uncommon for young children to suffer an intercondylar fracture of the distal humerus. Although many approaches have been described to manage, there is no specific and accepted treatment protocol for such fracture patterns. This study aimed to identify the incidence of intercondylar fracture of the distal humerus in the pediatric population and report the clinical outcome of external fixation and percutaneous pinning in such injury patterns.

**Methods:**

Pediatric patients under the age of 14 years who had an intercondylar fracture of the distal humerus treated with external fixation and percutaneous pinning between January 2013 and December 2018 at the author’s Wuhan Union Hospital were retrospectively evaluated. The detailed baseline information of the patients, operating time, time to union time, and carrying angle difference (CAD) of the injured extremity were collected. During the follow-up visit, clinical results were evaluated using the Mayo Elbow Performance Score (MEPS) and the Flynn criteria.

**Results:**

A total of eight patients (2 women and 6 men) with an average age of 8 years (5–12 years) who had an intercondylar fracture of the distal humerus (1 C2 and 7 C1) were included. All the patients achieved union, and the average MEPS score was 95 points 24 months after the surgery.

**Conclusion:**

The intercondylar fracture of the distal humerus in children is rare, and closed reduction and external fixation is a viable treatment option, especially for the C1 type of fracture pattern.

## Introduction

The intercondylar fracture of the distal humerus in children is considered to be a rare entity (1–10). Maylahn and Fahey reported an overall incidence of 6 (2%) among 300 elbow injuries in children (10). In this injury pattern, the medial and lateral condyles are often separated into independent fragments in a “T” or “Y” shape and lose contact with the humeral shaft causing rotational displacement.

In the past years, open reduction with internal fixation (ORIF) has been considered an effective treatment method for such fractures (8). Commonly reported surgical approaches are olecranon osteotomy, triceps-sliding, and triceps-splitting approaches. The most common short-term and long-term complications following ORIF are transient neuropathy (16.3%) and elbow stiffness (9.6%), respectively (9). With the recent trend toward the utilization of a minimally invasive approach in most surgical procedures, closed reduction with external fixation has been reported to provide satisfactory clinical results in pediatric fractures also (10, 11). So, most pediatric orthopedic surgeons have the discretion of using the closed method as much as possible.

This study aimed to identify the incidence of intercondylar fracture of the distal humerus in the pediatric population and report the clinical outcome of external fixation and percutaneous pinning after closed reduction in such injury patterns.

## Patients and Methods

Pediatric patients under the age of 14 years who had an intercondylar fracture of the distal humerus treated with external fixation and percutaneous pinning between January 2013 and December 2018 at the author’s Wuhan Union Hospital were retrospectively evaluated. All the surgeries were performed by a consultant pediatric orthopedic surgeon or under his direct supervision.

**FIGURE 1:**
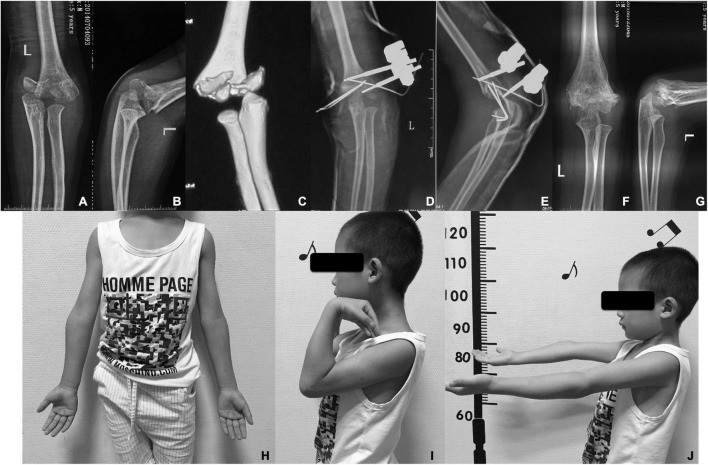
**(A)** Anteroposterior and **(B)** lateral radiographs of a 6-year-old boy with an intercondylar fracture of the distal humerus; **(C)** CT scan showing C1 type of AO classification; **(D)** anteroposterior and **(E)** lateral radiographs post-operation; **(F)** anteroposterior and **(G)** lateral radiographs at 12 months post-operation; and the follow-up in 24 months after surgery show excellent cosmetic results **(H)** and the functional appearance **(I,J)**.

The baseline information, including age, gender, and AO classification of fracture, was recorded preoperatively (Table 1). All fractures were diagnosed as per definition by the AO classification system relying on a radiograph or a CT scan (Figure 1). The postoperative data were collected during the follow-up visit. The clinical results were evaluated using the criteria of Mayo Elbow Performance Score (MEPS) (12) and Flynn degree (13). The authors assessing these patients’ clinical outcomes did not participate in the treatment. The Ethics Committee of the authors’ institute approved the study. Written informed consent was obtained from the legal guardians.

**TABLE 1:** Preoperative demographics of the patients.

No.	Age (years)	Gender	AO classification
1	7	W	C1
2	6	M	C1
3	10	M	C1
4	5	M	C1
5	7	W	C1
6	8	M	C1
7	9	M	C2
8	12	M	C1

### Surgical Technique

All the procedures were performed under general anesthesia. Initially, the first Schanz pin (2.7 or 3.0 mm) was inserted into the lateral condyle fragment distal to the physis under fluoroscopic guidance. The pin was placed parallel to the elbow joint and perpendicular to the longitudinal axis of the bone in order to avoid injuring the physis. The second Schanz pin was then inserted 2 cm proximal to the fracture line laterally and parallel to the first pin. This pin was tightly secured with the bicortical purchase, but great care was taken to avoid radial nerve injury. The lateral fragment was reduced with the proximal fragment by closed manipulation. After an acceptable reduction was achieved, the fracture fragments were held tightly together with clamps and rods. An anti-rotation K-wire (1.5–2 mm) was inserted in a retrograde fashion from the distal end of the lateral condyle and passed through the fracture line.

**FIGURE 2:**
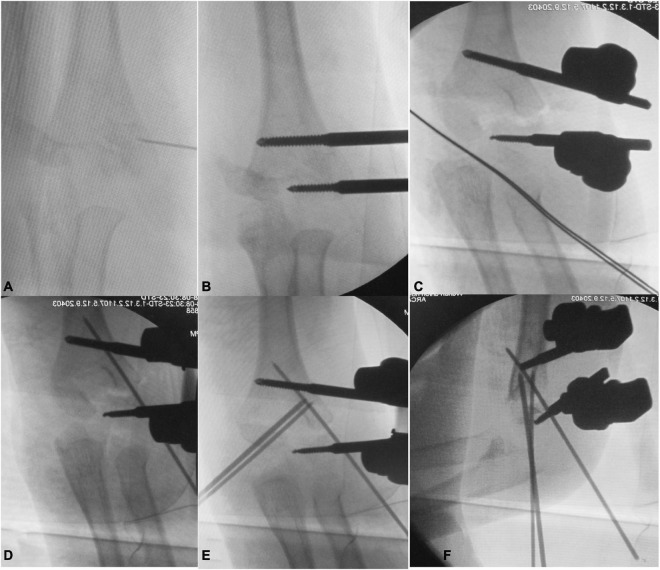
The C-arm x-ray during operation showed: **(A)** the distal lateral condyle fragment located with a syringe needle; **(B)** placement of radial unilateral external fixation; **(C)** reset the lateral side of the distal humerus by closed reduction and tightened external fixation; **(D)** placement of the radial side anti-rotation K-wire to stable the lateral fragment; **(E)** placement of the ulnar K-wire to stable the medial fragment; **(F)** lateral view of the elbow after fixation.

Another K-wire (1.5–2 mm) was inserted onto the distal medial condyle of the humerus, which acts as the joystick for the manipulation. After an acceptable alignment and reduction were achieved, the third K-wire was inserted from the medial condyle to the proximal fragment in a crisscross fashion. Then, the joystick pin was inserted further across the fracture line. The stability of the fixation and elbow movements were assessed in the anteroposterior (AP) and lateral views *via* fluoroscopy with gentle stress in maximum extension and flexion. The operated arm was immobilized with a posterior slab in a supine position with the elbow at 90° flexion (Operative stages are shown in Figure 2).

### Postoperative Care and Follow-Up

After the surgery, patients were discharged from the hospital once their condition allowed. The caregivers were taught to perform daily pin care. The plaster was removed after 3 weeks post-operation, and then, the child was allowed to start free elbow mobilization, but weight-bearing was avoided. AP and lateral radiographs of the operated elbow were taken at 3, 6, and 9–12 weeks and 6, 12, and 24 months. All the K-wire and external fixators were removed at 6 weeks in the outpatient visit. The weight bearing was allowed only after 12 weeks. The radiological union was considered once 3 out of 4 cortices were united (14), whereas radiological delayed union was considered if the visible gap were evident in 2 or more cortices at 12 weeks (14). The final clinical and radiological evaluations, including MEPS, Flynn criteria, CAD difference, and other complications, were performed at the last follow-up.

## Results

A total of 8 patients (2 women and 6 men) with an average age of 8 years (range, 5–12 years) were included in the study. According to the AO classification, 1 patient had a C2 type fracture and the rest of the other patients had C1 type fractures. Demographic details of the patients are shown in Table 1. The average duration of the surgery was 53.5 min (range, 46–60 min). All the fractures were clinically and radiologically united before 12 weeks (Table 2). At the last follow-up, all the patients showed satisfactory functional results on the MEPS score with an average of 95 points. All the patients’ carrying angle difference of the affected elbow was within 4 degrees, and they all showed good to excellent elbow function as per the Flynn scale (Table 2). Only two patients with superficial pin-site infection were identified during the follow-up visit, which resolved after 2–3 days of oral antibiotics. There were no non-union, neurovascular injury, myositis ossificans, or other surgery-related complications requiring further revision.

**TABLE 2:** Perioperative and follow-up data.

No.	OD (min)	FLT (month)	CAD	MEPS	UT (week)	Flynn
1	56	36	2	95	12	Excellent
2	60	37	3	95	10	Excellent
3	52	24	3	90	12	Good
4	55	48	2	95	9	Excellent
5	57	45	0	95	10	Good
6	49	27	4	95	10	Good
7	52	39	3	100	10	Excellent
8	46	42	3	95	12	Good

*OD, operation duration (min); FLT, follow-up time (month); CAD, carrying angle difference; MEPS, Mayo Elbow Performance Score; UT, union time.*

## Discussion

The most important finding of this study was that satisfactory fracture stability with acceptable postoperative outcomes could be achieved by external fixation and percutaneous pinning following a closed reduction in pediatric intercondylar humerus fracture.

There is no available consensus on the treatment of intercondylar fracture of the distal humerus in the pediatric population (1–11). Some surgeons insist that the open reduction and internal fixation is the ideal treatment for a pediatric T-condylar fracture of the humerus, which allows early elbow mobilization preventing stiffness (8, 10, 15). However, it cannot be denied that open reduction will bring more damage to the soft tissues and increase the risk of elbow stiffness (16–19). On the other hand, some authors advocate that the pediatric intercondylar fractures of the distal humerus can be treated with closed reductions and percutaneous pinning to obtain a satisfactory clinical outcome (16). Opinions vary as per the surgeon’s experience, but most surgeons accept that the goal of the treatment is to reconstruct the normal relationship of the joints and obtain good alignment.

To our knowledge, this is the first case series of pediatric humerus intercondylar fractures treated with external fixation and percutaneous pinning. Previously only the case report has been documented (20). The satisfactory result in our study may be attributed to most of the fractures (87.5%) in our series being the AO C 1 type. This type of fracture is a “T” shaped fracture with good bone quality where closed reduction can be performed successfully. As Ducic summarized, T-condylar fractures of the humerus are rare in children (21). A CT scan plays a significant role in the surgical plan in such a fracture pattern.

ORIF is an established surgical treatment method in adult and skeletally immature patients with intercondylar fractures of the humerus (21–24). However, surgical treatment for such fracture patterns in pediatric patients is controversial and has not been described in the literature. We adopted external fixation and percutaneous pinning, which led to a shorter duration of surgery and fracture union. Regardless of whether the patient population was subjected to olecranon osteotomy or triceps sparing surgery, the average duration of surgery was more than 77 min (21, 22). It is also worth emphasizing that there was only a negligible amount of bleeding in this series due to its minimally invasive nature. In the previous literature, the average time of fracture union following an ORIF was more than 11.5 weeks in previously published studies (21, 22), which is longer than the 10.6 weeks in this study. Similarly, 7 out of 8 patients had an MEPS score of the operated elbow of 95 points and above, and only 1 patient had an MEPS score of 90 points at the final follow-up, which is higher than the average MEPS score in other studies (21–23, 25). Compared to open reduction, closed reduction causes minimal damage to the skin and soft tissue, so the risk of postoperative joint stiffness is minimal (19). Percutaneous pinning after closed reduction is less invasive and does not increase the risk of complications. This technique may be an excellent alternative to open reduction for intercondylar fracture of the humerus (26). Due to the fact that there is minimal soft tissue and periosteal striping during surgery, the chance of bone healing is faster. The external fixator technology was initially proposed by Slongo (27), which has a fixation strength better than the simple K-wires providing sufficient stability to ensure early postoperative functional rehabilitation. Beck et al. (28) reported that early elbow Range of motion (ROM) following T-condylar fracture management produces a better final ROM with high patient satisfaction. Our technique provides better fracture stability allowing early ROM, resulting in better patient satisfaction. Another advantage of this technique is that the removal of the external fixation system can be completed in the outpatient setting and no secondary operation for implant removal is required (29).

The key points to remember for closed reduction and external fixation in patients with pediatric intercondylar fracture of the distal humerus are as follows: 1. Choosing an appropriate size Schanz pin: The surgeon should measure the size of the lateral condyle fragment in orthogonal x-ray views and then an appropriate sized (2.7 or 3.0 mm) Schanz pin should be inserted from the lateral condyle fragment parallel to the joint line and perpendicular to the longitudinal axis of the humerus under fluoroscopy guidance. 2. Avoiding nerve injury: there is always a chance of injuring the ulnar nerve during this procedure. The elbow should be placed in extension while inserting the K-wire in the medial condyle in order to avoid iatrogenic injury to the ulnar nerve.

Although the fracture pattern is rare in the pediatric population, this study still adopts the limitations of retrospective case series, such as a small sample size with no control group.

## Conclusion

The intercondylar fracture of the distal humerus in children is rare, and closed reduction and external fixation is a viable treatment option, especially for the C1 type of fracture pattern.

## Data Availability Statement

The raw data supporting the conclusions of this article will be made available by the authors, without undue reservation.

## Ethics Statement

The studies involving human participants were reviewed and approved by the Tongji Medical College, Huazhong University of Science and Technology (IORG No: IORG0003571). Written informed consent was obtained from the individual(s), and minor(s)’ legal guardian/next of kin, for the publication of any potentially identifiable images or data included in this article.

## Author Contributions

RZ, ZY, XL, and GJ involved in data collection and follow-up assessments. XT, SR, and HZ were responsible for the literature search and study design and finalized the manuscript. WS and RZ drafted the manuscript. All authors contributed to the article and approved the submitted version.

## Conflict of Interest

The authors declare that the research was conducted in the absence of any commercial or financial relationships that could be construed as a potential conflict of interest.

## Publisher’s Note

All claims expressed in this article are solely those of the authors and do not necessarily represent those of their affiliated organizations, or those of the publisher, the editors and the reviewers. Any product that may be evaluated in this article, or claim that may be made by its manufacturer, is not guaranteed or endorsed by the publisher.
